# Fermented soybean meal affects the ruminal fermentation and the abundance of selected bacterial species in Holstein calves: a multilevel analysis

**DOI:** 10.1038/s41598-020-68778-6

**Published:** 2020-07-21

**Authors:** Leila Kaviani Feizi, Sabihe Soleymanian Zad, Seyed Amir Hossein Jalali, Hassan Rafiee, Masoud Boroumand Jazi, Khaled Sadeghi, Rasoul Kowsar

**Affiliations:** 1 Department of Animal Science, College of Agriculture, Isfahan University of Technology, 84156-83111 Isfahan, Iran; 2 Department of Food Science and Technology, College of Agriculture, Isfahan University of Technology, 84156-83111 Isfahan, Iran; 3 Research Institute for Biotechnology and Bioengineering, Isfahan University of Technology, 84156-83111 10 Isfahan, Iran; 4 Department of Natural Resources, Isfahan University of Technology, 84156-83111 Isfahan, Iran; 5 Animal Science Research Department, Isfahan Agricultural and Natural Resources Research and Education Center, 81846-35745 Esfahan, Iran

**Keywords:** Bacterial systems biology, Systems analysis, Animal physiology

## Abstract

The effect of soybean meal (SBM) replacement with fermented SBM (FSBM) on ruminal fermentation and bacterial abundance in Holstein calves was investigated in this study. Thirty nine calves were randomized to: (1) control: 27% SBM + 0% FSBM (FSBM0, n = 13); (2) 18% SBM + 9% FSBM (FSBM9, n = 13); and (3) 13.5% SBM + 13.5% FSBM (FSBM13, n = 13). SBM contained a greater amount of large peptides containing 3 to 10 amino acids (AAs), while FSBM had a greater amount of ammonia nitrogen (NH_3_–N), free AAs, and small peptides containing 2 to 3 AAs. The calves fed FSBM13 had the lowest acetic acid, NH_3_–N, and the ratio of acetate to propionate, with the greatest concentration of caproic acid, valeric acid and isovaleric acid in ruminal fluid. Compared to those fed FSBM9 or FSBM13, the calves fed FSBM0 had the greatest proportion of *Butyrivibrio fibrisolvens* and *Ruminococcus albus* in rumen fluid. However, the ruminal abundance of *Prevotella ruminicola* in calves fed FSBM13 was greater than in calves fed FSBM0. Network analysis showed that the abundance of the *Ruminococcus albus* was associated with large peptides, and butyric acid was correlated with small peptide. Taken together, our findings suggest that FSBM may have the potential to boost calf performance by changing the fermentation products and the relative abundance of some members of the bacterial community in the rumen.

## Introduction

The calf starts its life with a physically and metabolically undeveloped rumen. Calves generally experience significant changes not only in the development of their rumen, but also in the rumen bacterial composition and community^1^. This, in turn, enables calves to digest dry feeds for their growth needs, impacting productivity and future milk production capacity^1,2^. Fibrolytic bacteria are important for the rapid anatomical and physiological growth of rumen, as the newborn calf moves from a liquid diet (i.e., milk) to a solid diet, such as grass and hay^3^.

Rumen microbes are classified into various functional groups, for example cellulolytic, amylolytic and proteolytic bacteria species^4^. These organisms metabolize feed components and other microbial products^5^*.* The rumen microbial community and the functional development of the stomach are affected by diets^4^. For example, when forages are fed to ruminants, *Bacteroidales* and *Ruminococcaceae* become dominant^4^. On the other hand, it is known that animals fed high-concentrate diets have more *Prevotella* and *Succinivibrionaceae* in their rumen fluid^4,6–8^. Therefore, diets fed to animals can alter the relative abundance of bacteria and the profile of volatile fatty acids (VFAs) in the rumen^1,4^. This, in turn, affects the evolution of the early ruminal development and microbiota^9^.

The chemical composition of the feed, such as peptides and amino acids (AAs), has been shown to alter in vitro and in vivo fermentation and the bacterial community in the rumen^10–12^. Carro and Miller^11^ reported that feeding peptides resulted in increased digestibility of fiber, VFA production, microbial nitrogen flow and microbial protein synthesis efficiency in calves. Fermentation is an efficient and cost-effective process that improves the quality of feed through microbial activity or microbial enzymatic action^13^. Soybean meal (SBM) proteins are extensively hydrolyzed to AAs and low molecular weight peptides due to microbial enzymatic activity and biochemical changes during fermentation with *Bacillus subtilis*^14,15^. Previous studies have shown that the fermentation of soya bean with *Bacillus subtilis* increases the proportion of A fraction; non-protein nitrogen is defined as the fraction A, which is metabolically closer to the soluble protein and consists of small peptides, free amino acids and NH_3_^16,17^. In addition, the amount of rumen undegraded protein (RUP) may increase in fermented soybean meal (FSBM) due to intensive heat treatment before fermentation (i.e., cooking)^18^. Protein fractions, such as RUP, have been reported to affect the performance and digestibility of calves^18,19^. Therefore, feeding fermented protein sources may affect the bacterial community, the production of VFAs and the growth of calves.

Previous studies have concentrated on the effects of FSBM on pig performance^20–23^, although few have studied the impact of FSBM on the fermentation of rumen and the bacterial community in dairy calves^24–26^. Our previous work has shown that feeding FSBM improved calf performance, increased starter intake and reduced weaning stress during abrupt weaning ^24^. Weaning stress has been known to reduce feed intake and calf performance during the immediate post-weaning period ^24–26^. Therefore, we hypothesized that fermentation may improve the protein fractions and peptide composition of SBM and affect ruminal fermentation and the abundance of some cellulolytic, fibrolytic and proteolytic bacteria in Holstein calves after abrupt weaning. Furthermore, we employed a multilevel approach, such as the hierarchical clustering analysis (HCA) and the principal component analysis (PCA), to determine the potential relationships between rumen fermentation and bacterial abundance.

## Results

### Protein fractions and peptide composition

As shown in Table 1, the protein content of SBM increased after fermentation from 43.3 to 48.2%. FSBM had greater levels of small peptides and free AAs, although it had lower levels of large peptides than SBM. The ammonia nitrogen (NH_3_–N), fractions A, B_3_, and C increased, although the fractions B_1_ and B_2_ decreased in FSBM compared to SBM. In comparison, RUP increased and rumen degraded protein (RDP) decreased in FSBM as compared with SBM. FSBM13 had greater amounts of crude protein, RUP, and fractions A, B3 and C, but had lower quantities of starch than FSBM0 and FSBM9.

**Table 1:** Protein fractions, peptide composition, and amino acid profile of soybean meal (SBM) and fermented SBM (FSBM).

Item	FSBM	SBM
Protein (% DM)	48.17 ± 0.44	43.30 ± 0.31
Large peptide (% of protein)^a^	4.96 ± 0.14	10.16 ± 0.65
Small peptide (% of protein)^b^	43.22 ± 0.38	40.11 ± 0.51
*Protein fraction (% of protein)*		
A (non-protein nitrogen)	13.78 ± 0.81	4.77 ± 0.26
B_1_ (rapidly degradable true protein)	1.87 ± 0.04	19.29 ± 0.95
B_2_ (moderately degradable true protein)	44.39 ± 1.25	65.89 ± 2.14
B_3_ (slowly degradable true protein)	28.40 ± 1.98	3.32 ± 0.19
C (undegradable true protein)	11.54 ± 0.49	6.71 ± 0.26
RUP	58.01 ± 1.75	38.35 ± 1.25
RDP	41.99 ± 1.25	61.65 ± 1.61
Phenylalanine	2.12 ± 0.04	1.77 ± 0.04
Proline	3.71 ± 0.17	3.12 ± 0.10
Histidine	2.75 ± 0.07	2.53 ± 0.11
Asparagine	4.98 ± 0.21	4.21 ± 0.21
Glutamine	9.19 ± 0.25	8.34 ± 0.15
Methionine	0.88 ± 0.05	0.75 ± 0.03
Lysine	2.96 ± 0.13	2.10 ± 0.15
Alanine	2.78 ± 0.16	2.14 ± 0.19
Serine	3.29 ± 0.22	2.56 ± 0.19
Leucine	4.01 ± 0.19	3.75 ± 0.26
Isoleucine	3.10 ± 0.28	2.24 ± 0.23
Tryptophan	2.74 ± 0.19	1.64 ± 0.08
Glycine	2.14 ± 0.14	2.11 ± 0.21
Cystine	0.71 ± 0.10	0.85 ± 0.09
Arginine	4.09 ± 0.32	4.15 ± 0.39
Threonine	2.34 ± 0.28	2.58 ± 0.11
Tyrosine	1.58 ± 0.12	1.68 ± 0.15
Valine	2.98 ± 0.20	2.51 ± 0.22
Total amino acids	56.35 ± 1.87	49.03 ± 1.70
Ammonia nitrogen (NH_3_–N, mg/g of dry mater)	2.25 ± 0.11	0.58 ± 0.04

Data were presented as mean ± SD; DM: dry matter.

^**a**^) Large peptide: consisting of 3–10 amino acids.

^**b**^) Small peptide: consisting of 2–3 amino acids.

### ***Concentrations of VFAs and ***NH_3_–***N in the rumen fluid of Holstein calves***

As there was a linear increase in the FSBM level (0, 9%, and 13.5%), a linear contrast analysis was performed (Table 2). A linear reduction in acetic acid concentration was found with an increase in the FSBM level (*P* = 0.04); calves fed FSBM13 had the lowest acetic acid concentration compared to those fed FSBM9 or FSBM0. Treatments did not affect the concentration of propionic acid (Table 2). This, in turn, resulted in a greater ratio of acetate to propionate in calves fed with FSBM0 (Table 2).

**Table 2:** Molar ratio of VFAs, concentration of NH_3_–N, and abundance of selected bacterial species in the rumen fluid of Holstein calves.

Items	Treatments ^a^			P value ^b^		
	FSBM0	FSBM9	FSBM13	Diet effect	Linear	Quadratic
*Rumen fermentation parameters*						
Acetic acid (Ac) (mol/100 mol)	52.42 ± 4.80^a^	36.48 ± 2.41^b^	35.38 ± 0.68^b^	**0.02**	**0.04**	0.31
Propionic acid (Pr) (mol/100 mol)	26.71 ± 3.17	21.57 ± 1.84	25.61 ± 1.71	0.27	0.65	0.14
Ac:Pr ratio	2.06 ± 0.11^a^	1.94 ± 0.21^ab^*	1.58 ± 0.12^b^	0.35	0.06	0.18
Isobutyric acid (mol/100 mol)	0.47 ± 0.04	0.46 ± 0.03	0.44 ± 0.02	0.61	0.53	0.98
Butyric acid (mol/100 mol)	4.73 ± 0.33	5.14 ± 0.21*	4.52 ± 0.14	0.74	0.55	0.11
Isovaleric acid (mol/100 mol)	0.50 ± 0.04^b^	0.57 ± 0.05^ab^*	0.71 ± 0.04^a^	**0.04**	**0.01**	0.52
Valeric acid (mol/100 mol)	1.36 ± 0.09^b^	1.46 ± 0.13^b^	2.04 ± 0.18^a^	**0.03**	**0.005**	0.19
Caproic acid (mol/100 mol)	0.34 ± 0.03^b^	0.65 ± 0.04^a^	0.67 ± 0.07^a^	**0.005**	**0.01**	0.15
NH_3_–N (mmol/l)	9.95 ± 0.33^b^	10.92 ± 0.36^ab^	12.03 ± 0.82^a^	**0.05**	**0.01**	0.25
*Rumen bacteria account (copies/ml)*						
*Ruminococcus albus,* × 10^9^	0.019 ± 0.001^a^	0.0002 ± 0.00001^b^	0.0001 ± 0.00005^b^	**0.02**	**0.04**	0.21
*Prevotella ruminicola,* × 10^9^	0.0006 ± 0.0001^b^	0.0088 ± 0.004^ab^	0.017 ± 0.007^a^	0.09	**0.05**	0.98
*Butyrivibrio fibrisolvens,* × 10^9^	0.0165 ± 0.001^a^	0.0028 ± 0.001^b^	0.0030 ± 0.001^b^	**0.0001**	**0.0001**	**0.0008**

Over time, calves fed various amounts of fermented soybean meal (FSBM). Calves were abruptly weaned at 59 days of age but remained in the experiment until day 67 when rumen fluid was collected (n = 13 calves per treatment).

^**a**^) FSBM0 (control, 27% SBM + 0% FSBM); FSBM9 (18% SBM + 9% FSBM); and FSBM13 (13.5% SBM + 13.5% FSBM).

^**b**^) The data was analyzed by the SAS software using the one-way ANOVA, followed by multiple comparisons tests, the Tukey’s test. The data was presented as Least Squares Means (LSM) ± Standard Error of the Mean (SEM).

Different superscripts (a, b) in a row indicate statistically significant differences at *P* < 0.05. * indicates *P* < 0.10: a tendency towards statistical significance between FSBM9 and FSBM13. Orthogonal polynomial contrasts were used to determine the linear or quadratic effect of FSBM using SAS software (SAS Institute Inc., Cary, NC).

^**c**^) The relative abundance of bacteria was measured using qPCR as a proportion of the total bacterial 16S rDNA.

The ruminal concentrations of NH_3_–N (*P* < 0.01), caproic acid (*P* < 0.01), valeric acid (*P* = 0.005) and isovaleric acid (*P* < 0.01) increased linearly in response to FSBM such that the FSBM13-fed calves had the greatest concentrations of NH_3_–N, caproic acid, valeric acid and isovaleric acid in rumen fluid (Table 2). Treatments did not affect the concentration of butyric acid and isobutyric acid in ruminal fluid (Table 2).

### *Detection of ruminal bacteria by p*olymerase chain reaction *(PCR)*

The results of the PCR products gel electrophoresis were shown in Supplementary Fig. 1. On the basis of the PCR assay, samples derived from fresh rumen fluid of cows were found to be positive for *Ruminococcus flavefaciens*, *Fibrobacter succinogenes*, *Ruminococcus albus, Butyrivibrio fibrisolvens*, and *Prevotella ruminicola* (Supplementary Fig. 1).

**Figure 1:**
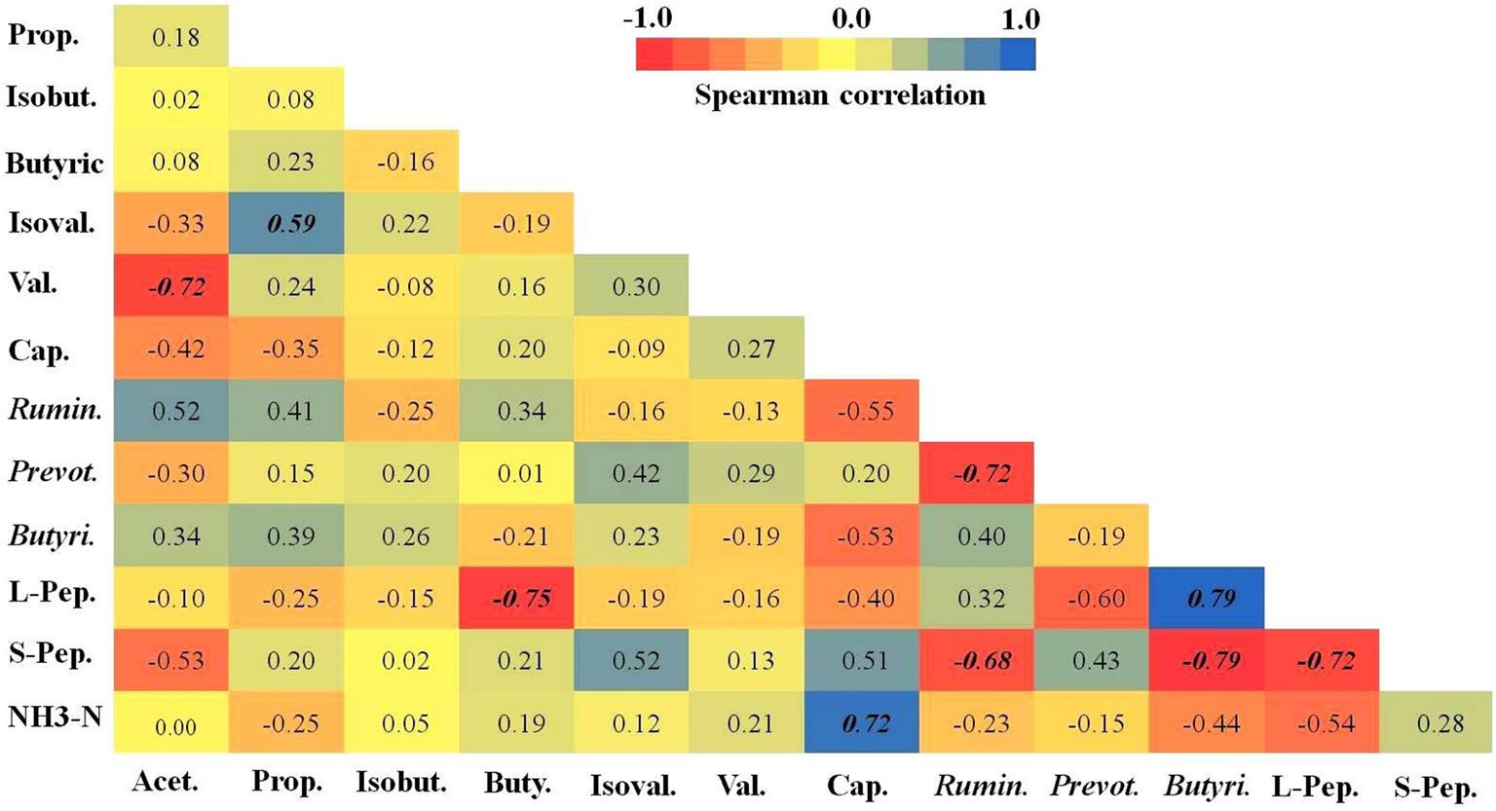
Pairwise-Spearman correlation between peptides, concentration of NH_3_–N, VFAs and bacterial abundance in the rumen fluid of Holstein calves. The color intensity is proportional to the correlation coefficients. The scale bar on the right side of the heatmap is the color range for different R values. Correlations that are significant at *P* < 0.05 are shown in the bold and italic profiles. It is important to note that the Spearman correlation can only determine that there is an association between two variables, so it does not imply a casual relationship^50^. Prop: propionic acid; Acet: acetic acid; Isobut: isobutyric acid; Isoval: isovaleric; Butyric: butyric acid; Val: veleric acid; Cap: caproic acis; Rumin: *Ruminococcus albus;* Prevot*: Prevotella ruminicola;* Butyri*:Butyrivibrio fibrisolvens;* L-Pep: large peptides; S-Pep: small peptides.

Next, PCR gel electrophoresis was conducted using experimental samples (calves). The PCR assay showed that the experimental samples (ruminal fluid collected from the claves) were found to be negative for *Ruminococcus flavefaciens* and *Fibrobacter succinogenes*. Samples were found to be positive for *Ruminococcus albus, Butyrivibrio fibrisolvens* and *Prevotella ruminicola.*

### qPCR for the determination of bacterial abundance in the rumen fluid of Holstein calves

The qPCR assay detected signals for all bacteria in the rumen fluid of cows (positive control). In relation to the total 16S rDNA gene copies per ml rumen fluid, the relative abundance of *Ruminococcus albus* (*P* = 0.04, linear) and *Butyrivibrio fibrisolvens* (linear, *P* = 0.0001, and quadratic, *P* = 0.0008) decreased with an increase in FSBM (Table 2). Compared with FSBM9- or FSBM13-fed calves, calves fed FSBM0 had the greatest abundance of *Ruminococcus albus* and *Butyrivibrio fibrisolvens* in relation to the total 16S rDNA gene copies per ml rumen fluid (Table 2). The relative abundance of *Prevotella ruminicola* (in relation to the total 16S rDNA gene copies per ml rumen fluid) increased linearly (*P* < 0.05) in response to an increase in FSBM. The calves fed FSBM13 had the greatest abundance of *Prevotella ruminicola* (in relation to the total 16S rDNA gene copies per ml rumen fluid) compared with calves fed FSBM0 (Table 2). The qPCR did not detect any signal for *Ruminococcus flavefaciens* and *Fibrobacter succinogenes* at Ct < 40; this threshold was set on the basis of the negative control Ct. The specificity of qPCR was shown in Supplementary Fig. 2.

**Figure 2:**
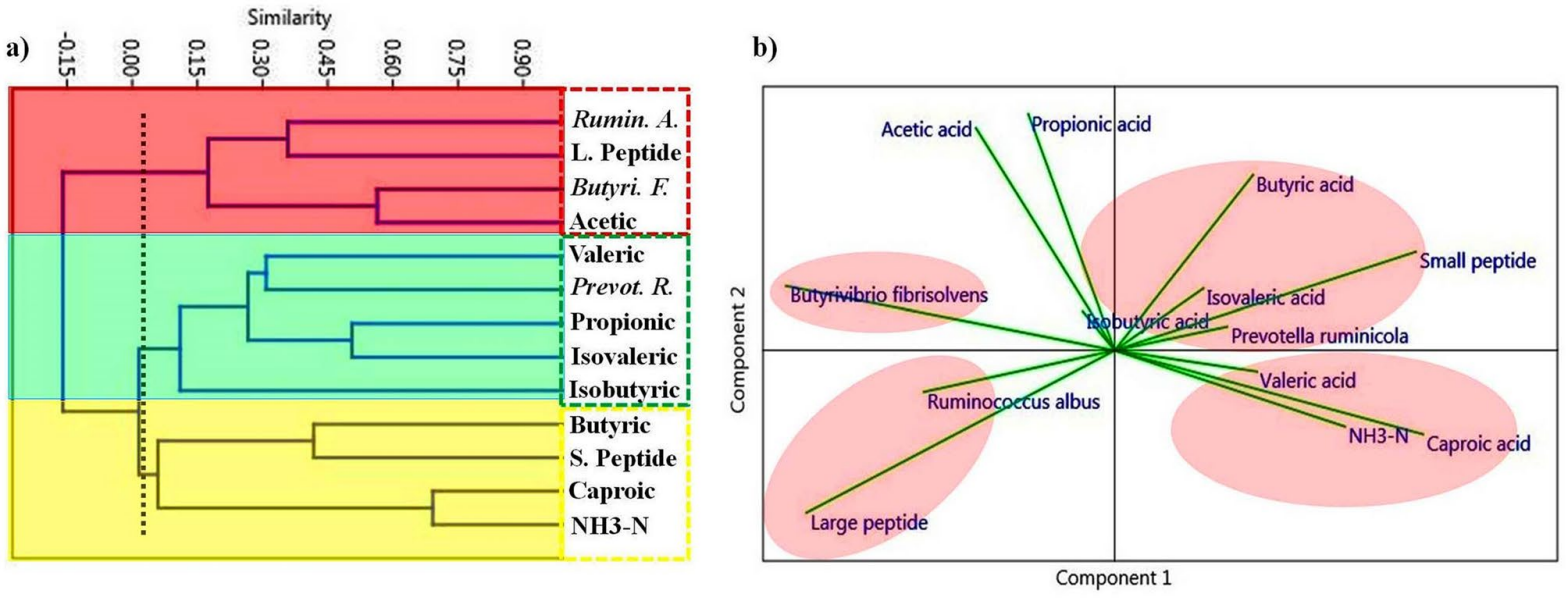
**(a)** Hierarchical cluster analysis (HCA) on peptides, ruminal VFAs, NH_3_–N and bacterial abundances. The HCA was developed by PAST using Spearman distance measurement and UPGMA. Boxes with different colors (i.e., red, green, and yellow) represent various clusters. **(b)** Biplot was developed from the principal component analysis of variables. Close-angles Vectors (< 45°) indicate a strong association, perpendicular vectors indicate no correlation, and vectors in opposite directions (approaching 180°) indicate a negative association. Rumin A: *Ruminococcus albus;* Butyri. F*: Butyrivibrio fibrisolvens;* Prevot. R*: Prevotella ruminicola;* L: large; S: small*.*

### ***Relationship between peptides and ruminal VFAs, NH***_***3***_***–N and bacterial abundance***

A Spearman correlation analysis was performed to determine the correlation between each pair of variables (Fig. 1). Significant correlations (*P* < 0.05) were observed between (1) acetic acid and valeric acid (R = − 0.72); (2) butyric acid and large peptides (R = − 0.75); (3) *Ruminococcus albus* abundance and small peptides (R = − 0.68); (4) *Ruminococcus albus* and *Prevotella ruminicola* abundance (R = − 0.72); (5) *Butyrivibrio fibrisolvens* abundance and small peptides (R = − 0.79). The concentration of NH_3_–N in rumen fluid was negatively correlated with *Ruminococcus albus* (R = − 0.23), *Butyrivibrio fibrisolvens* (R = − 0.44) and large peptides (R = − 0.54), but was positively correlated with small peptides (R = 0.28). In addition, there was a significant correlation between (1) the concentration of propionic acid and isovaleric acid (R = 0.59); (2) the concentration of NH_3_–N and caproic acid (R = 0.72); (3) the abundance of *Butyrivibrio fibrisolvens* and large peptides (R = 0.79) as shown in Fig. 1. The abundance of *Butyrivibrio fibrisolvens* was correlated with butyrate, its primary fermentation product (R = -0.21).

The HCA and PCA analyses were performed to clarify the concomitant interactions between peptides and ruminal VFAs, NH_3_–N and bacterial abundance. The HCA produced three separate clusters (Fig. 2a). The first cluster consisted of *Ruminococcus albus, Butyrivibrio fibrisolvens,* acetic acid, and large peptides (red box shown in Fig. 2a). The second cluster consisted of isovaleric acid, propionic acid, *Prevotella ruminicola,* isobutyric acid, and valeric acid (green box shown in Fig. 2a). The third cluster consisted of small peptides and ruminal butyric acid, caproic acid, and NH_3_–N (yellow box shown in Fig. 2a).

The PCA analysis revealed that the first four principal axis factors accounted for a sufficient amount of total variance (69.5%, Supplementary Table 1). The PCA plot showed that the proportion of *Ruminococcus albus* was positively associated with large peptides (directional vectors at < 45°; Fig. 2b). The proportion of *Ruminococcus albus* was negatively associated with *Prevotella ruminicola,* small peptides, butyric acid and isovaleric acid (directional vectors approaching 180°, Fig. 2b). In addition, small peptides were positively associated with ruminal *Prevotella ruminicola,* valeric acid, isovaleric acid, caproic acid and NH_3_–N (directional vectors at < 45°). However, ruminal NH_3_–N, valeric acid, and caproic acid were negatively associated with *Butyrivibrio fibrisolvens* (directional vectors approaching 180°)*.* PCA showed a negative association between *Butyrivibrio fibrisolvens* and butyrate, its main fermentation product (directional vectors approaching 180°, Fig. 2b)*.*

**Figure 3:**
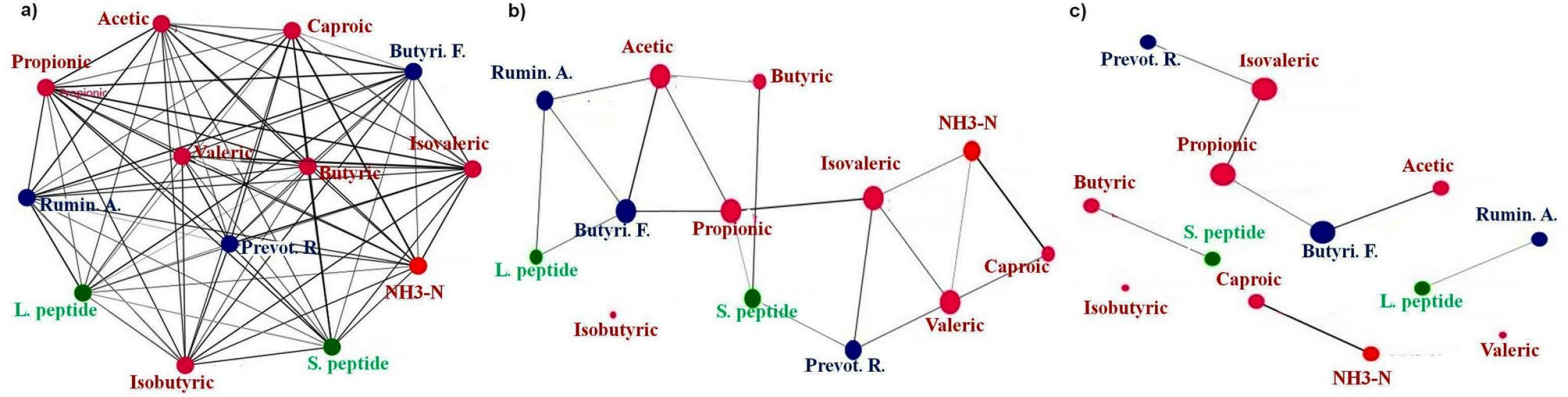
Network analysis to study the relationship between peptides, concentration of NH_3_–N, VFAs and bacterial abundance in the rumen fluid of Holstein calves. Network analysis and visualization was carried out by the PAST program using the Fruchterman-Reingold algorithm as a force-directed layout algorithm. The Rho correlation thresholds of (**a**) 0.0, (**b**) 50%, and (**c**) 60% were chosen for the description of edges (interactions between parameters) and nodes (variables). The threshold of 60% was the highest cut-off since there was no interaction between the variables afterwards. The size of the nodes and edges corresponds to the clustering and correlation coefficients, respectively. Small nodes and thin edges represent small values. Peptides are displayed in the green hue. The bacterium is shown in blue. The VFAs are displayed in red.

In the case of a 60% cut-off point, a correlation between acetic acid and *Butyrivibrio fibrisolvens* was found in a network analysis (darker line shown in Fig. 3c). This 60% cut-off point was selected as all parameters were disconnected from this level. Butyric acid was found to be associated with small peptides, while large peptides were linked with *Ruminococcus albus* (darker line shown in Fig. 3c). Caproic acid was also found to be associated with ruminal NH_3_–N. The network analysis confirmed the HCA and PCA findings, thus providing a better picture of the interactions between dietary peptides and ruminal NH_3_–N, VFAs and bacterial abundance.

## Discussion

In this study, the fermentation of SBM increased the total protein content and modified the protein fractions. The fermentation of SBM has been reported to increase the protein content of SBM from 43 to 49%^15,20,21^. The fermented SBM provides approximately 10% more protein than the traditional SBM^18^. Potentially, an increase in the production of microbial proteins during fermentation may explain the greater protein content of FSBM^15,27^. It was also found that free AAs, small peptides, and fractions A (non protein nitrogen) increased in FSBM, in agreement with other studies^15,16,22,25–27^. This may be the result of proteolytic activity of microorganisms (such as *Bacillus* spp.) and the release of proteolytic enzyme during fermentation^16,18,28^. Weng and Chen^28^ found that the concentration of small peptides in soybean increased after 24 h of fermentation. In the present study, the fermentation time for SBM was 48 h, which may explain the greater percentage of fraction A (which consists of free AAs and small peptides^16,17^) in FSBM.

Zhang et al.^29^ found that small peptides had a positive effect on nitrogen retention and the use of protein in goats. In addition, Cao et al.^30^ reported that, despite digesting greater amounts of nitrogen than those fed SBM, soybean peptide-fed sheep had smaller losses of nitrogen from the feces and urine. However, Wang et al.^31^ demonstrated a decrease in NDF and ADF digestibility due to infusion of soybean small peptides. It should be remembered that, due to greater amounts of small peptides and RUP, the feeding of FSBM13 may compromise the digestibility of the fiber and the concentration of acetate in calves. In response to RUP, Griswold et al.^32^ observed a decrease in total concentrations of VFAs, acetate and butyrate in continuous culture. It seems that by improving the metabolism of nitrogen, the use of FSBM with greater small peptides has led to an increase in the growth performance of FSBM13-fed calves reported in our previous study^24^.

Importantly, it should be noted that FSBM was prepared by incubation of SBM with *Bacillus subtilis*, which has been shown to have probiotic effects. Hence, *Bacillus subtilis* may enter the rumen and alter the microbial composition, fermentation and digestive processes of the rumen^33,34^. For example, Zhang et al.^33^ found that the probiotic bacteria (a mixture of *Lactobacillus plantarum* and *Bacillus subtilis*) influenced the rumen bacterial community and reduced the numbers of cellulolytic bacteria such as *Ruminococcus albus*. Therefore, further research with killed colonies (i.e., sterilized FSBM) is required to distinguish the role of peptide size and *Bacillus subtilis* (as a probiotic bacterium) in rumen physiology. In addition, an investigation into the presence of these bacteria in the rumen (i.e., qPCR) may help to explain the possible probiotic effect of *Bacillus subtilis*.

As described above, fermentation increased small peptides and free AAs in FSBM, resulting in an increased fraction A, consisting mostly of free AAs and small peptides^35,36^. In addition, thermal administration during fermentation tends to be capable of transforming fraction B2 into fractions B_3_ (slowly degradable true protein) and C (undegradable true protein)^35^. Toti et al.^37^ reported that heat treatment decreases fraction B1, but increases B3 and C fractions in canola meal.

The present results revealed a decline in the ruminal concentration of acetic acid in calves fed FSBM13 or FSBM9. Similarly, several studies have reported that feeding peptides or SBM small peptides reduces the concentrations of acetic acid in the rumen^31,38^. The decreased concentration of acetic acid in calves fed FSBM13 seems to be partially due to a decrease in the abundance of *Ruminococcus albus* (one of the fiber digestive bacteria)^39,40^ and *Butyrivibrio fibrisolvens* in the rumen^41^. In particular, network, Spearman correlation, PCA, and HCA analyses showed that acetic acid was associated with *Butyrivibrio fibrisolvens*, suggesting that feeding FSBM13 can result in a decrease in both acetic acid and *Butyrivibrio fibrisolvens* abundance in calves. It is important to note that during the entire study and at the time of the collection of rumen fluid (day 67), calves were fed experimental starters with 21–23% NDF (almost medium-NDF levels) and 10% chopped alfalfa hay. The use of straw bedding by calves during the experiment was found to be very rare. It should also be noted, as a limitation of this study, that the stomach tube was used in this study to collect rumen fluid. This method mainly collects the liquid parts of rumen, not the solid particles of rumen. Many bacterial species, particularly cellulolytic bacteria, are strongly attached to solid particles^39,40,42^. These limitations might explain the low abundance of *Butyrivibrio fibrisolvens* (a fibrolytic bacterium) and *Ruminococcus albus* (a cellulolytic bacterium). Moreover, no signals were found in this study for *Ruminococcus flavefaciens* and *Fibrobacter succinogenes* in experimental samples (i.e., calves) using qPCR. However, positive signals for these bacteria were detected in the fresh rumen fluid of cows. Shinkai and Kobayashi^42^ did not detect one group of *Fibrobacter succinogenes* in the rumen content of sheep by qPCR test. They reported that the fluorescence signals from rumen fluid were mainly weak, and the abundance of *Fibrobacter succinogenes* in rumen fluid was considerably smaller than in rumen-incubated hay samples^42^. It appears that a large number of cellulolytic bacteria were not obtained due to the poor efficiency of the stomach tubes used in this study. As a result, the present results did not cover the full spectrum of the rumen bacterial population; further research is required to identify the effect of FSBM on the rumen bacterial communities in calves.

The increase in FSBM resulted in greater concentrations of valeric acid, isovaleric acid and caproic acid. Both *Ruminococcus albus* and *Butyrivibrio fibrisolvens* species are the main consumers of branched-chain VFAs (BCVFAs, e.g. isovaleric acid)^43^. Therefore, the decreased abundance of these bacteria might explain greater concentrations of isovaleric acid in FSBM13-fed calves. In fact, BCVFAs are primarily derived from ruminal digestion of dietary proteins^44^. Hence, the greater concentrations of isovaleric acid and valeric acid in FSBM13-calves can be explained by the greater amount of crude protein and small peptides in FSBM than in SBM. Moreover, the Spearman correlation demonstrated a positive, but not significant, correlation between small peptides and ruminal concentrations of isovaleric acid and valeric acid. In addition, there was a positive correlation between *Butyrivibrio fibrisolvens* and isobutyrate, although there was no significant relationship between cellulolytics and isobutyrate. Previous studies have shown that BCVFAs improve the apparent digestibility of dry matter, microbial growth, microbial function, and enzyme activity in ruminants^45,46^. We also recently reported an improvement in the performance of FSBM13-fed calves compared to SBM-fed calves^24^. These findings suggest that the feeding of FSBM may alter the rumen conditions for the growth of the calves.

Data revealed that FSBM13 increased *Prevotella ruminicola* but decreased the abundance of *Ruminococcus albus* and *Butyrivibrio fibrisolvens*. Wang et al.^31^ reported that the infusion of soybean small peptides had no effect on abundance of *Prevotella ruminicola* and *Ruminococcus albus* in cows. The data revealed a negative correlation between *Butyrivibrio fibrisolvens* abundance and small peptides, while Wang et al.^31^ reported an increase in *Butyrivibrio fibrisolvens* abundance in cows in response to the infusion of soybean small peptides. This implies that other factors of SBM/FSBM or fermentation (i.e., *Bacillus subtilis*) may have an effect on the composition of the bacteria in the rumen fluid, in addition to small peptides.

Generally, the primary fermentation products of *Ruminococcus albus*, *Butyrivibrio fibrisolvens* and *Prevotella ruminicola* are acetate, butyrate and propionate, respectively^38,47^. The greater abundance of *Ruminococcus albus* and *Butyrivibrio fibrisolvens* appears to result in greater concentrations of acetic acid in FSBM0-calves. As shown by the fact that these two strains together account for only a small portion of the total bacterial population, several other species may have been responsible for the bulk of the increase in acetic acid as their main fermentation product.

Interestingly, this was discovered by the PCA, *Butyrivibrio fibrisolvens* was adversely associated with butyrate, its main fermentation product^47^, but the cause remains uncertain. Nevertheless*, Butyrivibrio fibrisolvens* have been found to be substantially associated with butyrate in the subacute rumen acidosis model^48^. The Spearman analysis also revealed that *Butyrivibrio fibrisolvens* had a negative association with the concentrations of NH_3_–N in rumen. Sales et al.^49^ reported that, the proteolytic activity of *Butyrivibrio fibrisolvens* increased as a reaction to NH_3_–N, while the proteolytic activity of *Prevotella albensis* decreased. They proposed that each species would follow its own dietary strategy to conform to the environmental conditions of the rumen ecosystem^49^. The inverse association between *Butyrivibrio fibrisolvens* and butyrate or between *Butyrivibrio fibrisolvens* and NH_3_–N may be part of the regulatory feedback within the rumen microbial ecosystem; more research is required to validate this hypothesis. It is important to note that the Spearman correlations can only indicate whether there is an association between two variables, and this does not mean a casual relationship^50^.

The PCA showed that large peptides had a strong association with the abundance of *Ruminococcus albus* and *Butyrivibrio fibrisolvens*. Regarding variations in FSBM levels, fermentation and bacterial abundance in rumen may vary depending on the size of the peptide. As described above, the potential role of *Bacillus subtilis* in rumen physiology needs to be investigated in order to differentiate the effects of peptide size and *Bacillus subtilis* on rumen bacterial community.

Calves fed FSBM13 had a greater proportion of *Prevotella ruminicola* in the rumen fluid. This proteolytic bacterium effectively consumes NH_3_–N, peptides and, to a lesser degree, uses AAs to produce microbial proteins and adenosine triphosphate (ATP)^51,52^. The PCA showed that *Prevotella ruminicola* was positively associated with the concentration of NH_3_–N in rumen. *Prevotella ruminicola* has been reported to grow faster and to a greater final dry weight in the presence of soybean protein than casein^53^. Hence, feeding FSBM13 containing small peptides and free AAs might contribute to increased growth of proteolytic *Prevotella ruminicola* to reduce the high NH_3_–N levels^52^.

In summary, proteolytic *Prevotella ruminicola* responded positively to FSBM, while other cellulolytic/fibrolytic bacteria, such as *Ruminococcus albus* and *Butyrivibrio fibrisolvens*, reacted negatively. On the basis of the present findings and our previous research^24^, it can be concluded that feeding FSBM will improve the growth performance and starter intake of Holstein calves by changing the fermentation and abundance of some bacteria in the rumen. More studies with both liquid and solid phases of the rumen content and a broader variety of bacteria are needed to provide a deeper understanding of the effects of FSBM on the rumen ecosystem.

## Methodology

### Animal studies and data statement

Animal experiments were conducted in accordance with the Guiding Principles for the Care and Use of Research Animals developed by the Isfahan University of Technology, Iran. The protocol and methods were approved by the Committee on the Ethics of Animal Experiments of the Isfahan University of Technology (No. 390132). The datasets are available from the corresponding author upon request.

### Animals, experimental design and diets

Thirty-nine Holstein calves with an average initial body weight of 39.5 ± 3.3 kg were assigned to 3 treatments (7 male and 6 female per treatment) from birth until 67 days of age. The calves were separated from their dams immediately after birth, weighed, and moved to individual hutches (1 × 1.5 m) bedded with straw, which was renewed every 24 h. The calves were housed in individual hutches during the entire study (67 days). The use of straw bedding by calves was also checked and was found to be very rare during the experiment.

Calves received 2.5 L of colostrum in the first 1.5 h of life, followed by 5 L of colostrum in the first 12 h of life. By the third day, colostrum and transitional milk were given to calves. From day 4, whole milk (5 L per d, temperature: 39 ± 2 °C) was fed to calves using a plastic bucket at 0,040 and 1,600 h. The average milk composition used during the trial was 3.30 ± 0.13% fat, 2.85 ± 0.01% crude protein, 4.93 ± 0.06% lactose, and 11.00 ± 0.52% total solids (mean ± SD). During the entire study, the calves had free access to water and the ad libitum starter intake (which included different quantities of SBM or FSBM) was accomplished by providing a quantity that resulted in a residue of 10% of the feed offered after 24 h. The feed refusal for each individual calf was collected at 0,800 h. From the first day of life, a starter was provided mixed with chopped alfalfa hay until day 67 of the experiment. During the entire study (67 days) and at the time of the collection of rumen fluid (day 67), calves were fed experimental starters. All calves were suddenly weaned by removing the milk meal at 8 weeks of age, but remained in individual hutches until the end of the experiment (day 67).

Experimental treatments were (1) 27% SBM + 0% FSBM (FSBM0, the control group); (2) 18% SBM + 9% FSBM (FSBM9); and (3) 13.5% SBM + 13.5% FSBM (FSBM13). All diets were isoenergetic and formulated according to the Cornell Net Carbohydrate and Protein System (CNCPS, version 5.1). The ingredients and chemical compositions of the experimental diets are shown in Table 3.

**Table 3:** Ingredients and chemical composition (% of dry matter, unless otherwise stated) of experimental starter feeds.

Items	Treatments^a^		
	FSBM0	FSBM9	FSBM13
*Ingredients, % of dry matter*			
Chopped alfalfa hay	10	10	10
Barley grain, ground	26.6	26.6	26.6
Corn grain, ground	26.6	26.6	26.6
Soybean meal	27	18	13.5
Fermented soybean meal	0	9	13.5
Wheat bran	6.8	6.8	6.8
Vitamin and mineral mix^b^	1.00	1.00	1.00
Calcium carbonate	0.75	0.75	0.75
Di calcium phosphate	0.19	0.19	0.19
Sodium bicarbonate	0.70	0.70	0.70
Salt	0.36	0.36	0.36
*Chemical composition, % of dry matter*			
ME (Mcal/kg)^c^	2.70	2.71	2.71
NE_g_ (Mcal/kg) ^**c**^	1.54	1.54	1.55
DM	90.48 ± 0.51	90.90 ± 1.11	91.09 ± 1.87
OM	92.17 ± 0.18	92.09 ± 1.14	92.39 ± 1.32
CP	19.72 ± 0.24	20.16 ± 0.26	20.38 ± 0.14
Ether extract	3.01 ± 0.11	3.12 ± 0.09	3.63 ± 0.14
NDF	23.86 ± 0.95	23.13 ± 1.10	21.30 ± 0.97
ADF	8.33 ± 0.47	8.79 ± 0.68	8.11 ± 0.73
NFC ^**d**^	45.58 ± 1.21	45.68 ± 1.14	47.08 ± 1.26
Starch	34.14 ± 1.11	33.55 ± 2.02	33.04 ± 1.36
RUP	8.12 ± 0.24	8.61 ± 0.16	8.92 ± 0.74
RDP	11.6 ± 0.16	11.20 ± 0.54	10.79 ± 0.91
A (non-protein nitrogen)	3.86 ± 0.22	4.02 ± 0.54	4.13 ± 0.32
B_1_ (rapidly degradable true protein)	1.94 ± 0.11	1.67 ± 0.15	1.54 ± 0.19
B_2_ (moderately degradable true protein)	10.78 ± 0.41	10.68 ± 0.65	10.55 ± 0.87
B_3_ (slowly degradable true protein)	2.28 ± 0.13	2.80 ± 0.24	3.07 ± 0.19
C (undegradable true protein)	0.88 ± 0.05	1.02 ± 0.10	1.21 ± 0.11
Ca ^**c**^	0.67	0.66	0.66
P ^**c**^	0.53	0.53	0.53

^**a**^) FSBM0 = 27% soybean meal + 0% fermented soybean meal, FSBM9 = 18% soybean meal + 9% fermented soybean meal, FSBM13 = 13.5% soybean meal + 13.5% fermented soybean meal.

^**b**^) Contained per kilogram of supplement: 250,000 IU of vitamin A, 50,000 IU of vitamin D, 1,500 IU of vitamin E, 2.25 g of Mn, 120 g of Ca, 7.7 g of Zn, 20 g of P, 20.5 g of Mg, 186 g of Na, 1.25 g of Fe, 3 g of S, 14 mg of Co, 1.25 g of Cu, 56 mg of I, and 10 mg of Se.

^**c**^) Calculated according to NRC (2001).

^**d**^**)** NFC = 100—% neutral detergent fiber—% crude protein—% ether extract—% ash**;** Data are presented as mean ± SD.

### Fermentation of SBM

The FSBM was developed by a commercial company (Mehre Bisotun Co., Isfahan, Iran) as described by others^27^. The method used to prepare the FSBM was detailed in our previous work^24^. In brief, the dried SBM (90% dry matter) was immersed in distilled water (385 g water/1,000 g SBM) for 60 min, taking the moisture content of SBM to about 35%. The hydrated SBM was then cooked in a steam tank at 65 °C for 1 h and allowed to cool to room temperature. The cooled SBM was then inoculated with *Bacillus subtilis* GR-101 (4 log cfu/g of SBM), mixed and fermented in a bed-packed incubator for 48 h. After fermentation, the FSBM was dried at 50 to 60 °C to reduce its moisture content to around 10% before milling with a hammer mill. The protein fractions, peptide composition, and AAs profile of SBM and FSBM are shown in Table 1.

### Data collection, sampling procedures, gas chromatography, and NH_3_–N determination

Chemicals and media were from Sigma Chemical Co. (St. Louis, MO) and Gibco (Grand Island, NY, USA), unless otherwise mentioned. Approximately 20 ml of rumen fluids was collected from all calves (n = 13 calves per treatment) one week after weaning (day 67) at 1,100 h, four hours after feeding in the morning. Rumen fluid samples were collected using a stomach tube (a flexible polyvinyl chloride tube with 2 mm of wall thickness, 145 cm long, 8 mm internal diameter, and with 20 holes of 3 mm diameter in the 12-cm probe end, Cristallo Extra, FITT S.p.A., Sandrigo, Italy) and an electric vacuum pump (down to 8–9 mbar, Fiam Motor, Iran). The first 20 ml of rumen contents was discarded to avoid contamination with saliva. In addition, samples with abnormal viscous appearance were deemed to have been contaminated with saliva and discarded. Rumen fluid samples were immediately filtered through four layers of cheese cloth and divided into two parts. The first portion (10 ml) was mixed with 2 ml of 250 g/l (w/v) metaphosphoric acid and preserved at − 20 °C for VFAs analysis. The second part of the rumen fluid (10 ml) was immediately centrifuged at 9,000 × *g* for 10 min. The supernatants were discarded and the resulting pellets were resuspended into a microtube containing 1 ml of the RNAlater solution (Life Technologies, Gaithersburg, MD, USA) and stored at –80 °C for DNA extraction and qPCR.

The concentrations of VFAs were measured using a capillary column (SGE DBX5, length: 30 m, inside diameter: 0.25 mm; with a 25 micron film; Varian CP-WAX 52 CB, Varian Inc., Palo Alto, CA) on a Varian 3,900 gas chromatograph equipped with a flame ionization detector. The temperature of the injector and detector was 230 °C and 240 °C, respectively. Nitrogen was used as a carrier gas with a flow rate of 1.8 ml min^−1^.

The concentration of ruminal NH_3_ was measured by a spectrophotometer (Ultramicroplate Reader, ELx808, Bio-Tek Instruments, Inc., Winooski, VT, USA) using a colorimetric method based on the reaction of NH_3_ with hypochlorite and then with phenol^54^.

### Cultivation and isolation of anaerobic bacteria

In order to provide microbial DNA, anaerobic bacteria were cultivated and isolated using fresh rumen fluid from cows. The microbial DNA was used to prepare the qPCR standard curve and to test the integrity of the cDNA. Also, in the event that these samples (fresh rumen fluid from cows) were found to be positive for the target bacteria (using PCR gel electrophoresis), these samples were used as a positive control for the qPCR.

Fresh rumen fluid (50 ml per each replication) was collected from the ruminally cannulated lactating Holstein cows for microbial cultivation. Rumen fluid samples were placed in a plastic bottle previously flushed with CO_2_ using an oxygen absorber-CO_2_ generator (GasPak, AnaeroPack sachet, Mitsubishi Gas Chemical America, Inc.) to protect it from air^55^. Fresh rumen fluid samples were immediately transported to the laboratory within 30 min using an insulated container and cultivated under anaerobic conditions. Hungate’s roll-tube anaerobic technique^56^, modified by Bryant and Burkey^57^, was used for the cultivation of anaerobic bacteria. The anaerobic culture medium used was based on the rumen fluid-glucose-cellobiose-agar (RGCA) composition as follows: 1,000 ml of culture medium contains 399.5 ml of fresh rumen fluid, 216.5 ml of solution IV (including 2.0 g of glucose, 2.0 g of cellobiose, 14.98 g of agar), 149.8 ml of mineral solution I (including 0.3% K_2_HPO_4_), 149.8 ml of mineral solution II (including 0.6% (NH_4_)_2_SO_4_, 0.6% NaCl, 0.3% K_2_HPO_4_, 0.06% MgSO_4_, 0.06% CaCl_2_), 66.7 ml of 6% Na_2_CO_3_ solution, 16.7 ml of 3% cysteine hydrochloride solution, and solution III (1 ml of 0.1% resazurin). Cysteine hydrochloride was used to reduce the culture media prior to inoculation^55^. In addition, adequate concentrations of Na_2_CO_3_ were added to the culture media in order to maintain the pH of culture at 5.6–5.8. This mixture was put in a dilution bottle and sterilized with an autoclave at 120˚C for 20 min. Next, the solution was allowed to cool about 45˚C and then bubbled with O_2_-free CO_2_ gas until resazurin indicator changed from pink to colorless. After solidifying the culture media using agar (2% wt/vol), RGCA (4 ml) was put in a roll tube, autoclaved at 120˚C for 5 min, and held in a water bath at 45˚C for 30 min. Upon inoculation, the tubes were gently rolled in cold water to form a thin layer of rumen fluid-agar around the inside.

### Microbial DNA extraction and real-time polymerase chain reaction

Microbial DNA was extracted from rumen samples (pellets derived from the experimental rumen fluid of the calves) and from total bacterial colonies (derived from the fresh rumen fluid collected from cows to provide positive control). The extraction was fully mixed (2 mg) with 500 μl of cetyltrimethyl ammonium bromide (CTAB) lysis buffer (composed of 2% CTAB, 100 mmol Tris–HCL, 25 mM EDTA and 2 mol NaCl) and incubated at 65 °C for 15 min according to the CTAB method^58,59^. The tubes were then centrifuged at 14,000 × g for 5 min at 4 °C, and the resulting supernatant was transferred to 2 ml microcentrifuge tube containing 300 μl of chloroform: isoamyl alcohol (24:1). The mixture was centrifuged at 14,000 × g for 5 min at 4 °C, then precipitated with 200 μl of ethanol 70%, centrifuged at 14,000 × g for 5 min at 4 °C and allowed to air-dry at room temperature. The resulting pellets were collected and dissolved in a Tris–EDTA buffer (100 μl) to dissolve DNA; the extracted DNA was stored at − 80 °C for further use^58,59^. In the event that samples (from cows) were found to be positive for the target bacteria using PCR product gel electrophoresis, microbial DNA was extracted from these samples and used as positive control for the qPCR assay.

In this study, three cellulolytic bacteria, including *Ruminococcus albus, Ruminococcus flavefaciens, Fibrobacter succinogenes*, and two proteolytic bacteria, including *Butyrivibrio fibrisolvens* and *Prevotella ruminicola*, were qPCR-tested species. The abundance of bacteria in rumen fluid was measured using qPCR as a proportion of the total bacterial 16S rDNA based on the following formula^60–63^:

$${\text{Relative}}\;{\text{quantification}} = 2^{{ - ({\text{Cycle}}\;{\text{threshold }}\left[ {Ct} \right]{\text{target }} - {\text{ Ct}}\;{\text{total}}\;{\text{bacteria}})}}$$

The Cycle threshold (Ct) was the number of cycles needed for the fluorescent signal to exceed the threshold. The method used to compare the relative expression data in real-time PCR was the delta-delta Ct (2^–ΔΔCt^) method, relative quantification without efficiency correction^64^; since this method assumes that the target DNA is optimally doubled during each real-time PCR cycle^64^.

The primers used for qPCR and their efficiency are shown in Table 4. The efficiency of the qPCR reaction was sufficient, on average 99.68 ± 2.71 (mean ± SD, Table 4). The results were omitted where Ct values of the samples were greater than Ct of the corresponding negative control (i.e., > 40). In the case of *Ruminococcus flavefaciens* and *Fibrobacter succinogenes*, the Ct values of qPCR (for all samples derived from rumen fluid of experimental calves) were greater than Ct of the corresponding negative control. All real-time qRT-PCR reactions were triplicated and conducted on a StepOne Plus™ real time PCR device (Applied Biosystems, Life Technologies, Massachusetts, USA). The reaction mixture (10 μl) consisted of 5 μl Master mix, 1 μl of each primer (10 pmol/μl), 0.2 μl of 50 × ROX Reference Dye (Invitrogen, Madrid, Spain ×), 1.8 μl ddH_2_O and 1 μl of extracted DNA.

**Table 4:** Bovine primers used in qPCR.

Bacteria		Sequence of nucleotide (5′–3′) *	Tm, °C	Ry^2^	Slope	Efficiency, %	**Accession no**	**Ref**
*Ruminococcus*	F	CCCTAAAAGCAGTCTTAGTTCG	60	0.992	− 3.394	97.8	CP002403.1	62,63
*albus*	R	CCTCCTTGCGGTTAGAACA						
*Ruminococcus*	F	CGAACGGAGATAATTTGAGTTTACTTAGGTTAGG	58.5	0.987	− 3.331	98.9	AB849343.1	62
*flavefaciens*	R	CGGTCTCTGTATGTTATGAGGTATTACC						
*Fibrobacter*	F	GTTCGGAATTACTGGGCGTAAA	59.5	0.985	− 3.356	102.5	AB275512.1	62
*succinogenes*	R	CGCCTGCCCCTGAACTATC						
*Butyrivibrio*	F	TCTGGAAACGGATGGTA	55	0.994	− 3.256	102.1	HQ404372.1	62
*fibrisolvens*	R	CCTTTAAGACAGGAGTTTACAA						
*Prevotella*	F	GAAAGTCGGATTAATGCTCTATGTTG	58	1.00	− 3.421	95.6	LT975683.1	62
*ruminicola*	R	CATCCTATAGCGGTAAACCTTTGG						
Total bacterial	F	CGGCAACGAGCGCAACCC	60	0.996	− 3.324	101.2	AY548787.1	61,62
16S rDNA	R	CCATTGTAGCACGTGTGTAGCC						

The efficiency of the qPCR reaction was sufficient, on average 99.68 ± 2.71 (mean ± SD). *F* Forward, *R* Reverse.

The microbial DNA used for standard curves was directly extracted from the cultivated colonies of cow rumen fluid. In brief, the standards for each real time PCR test were developed using the regular PCR. Then, the PCR product was purified using a QIAquick PCR purification kit (Qiagen, Valencia, CA, USA). Decimal DNA standard dilutions were used to plot the standard curve for each primer pair used. The qPCR was conducted under the following conditions: 95 °C for 10 min, 95 °C for 30 s followed by 60 °C for 30 s, 72 °C for 20 s, and 60 °C for 1 min for 42 cycles. For each assay, a negative control without a cDNA template was used.

### Quality control of DNA extracts and polymerase chain reactions

In order to test the integrity of cDNA, PCR products gel electrophoresis was conducted on a number of fresh rumen fluid samples obtained from lactating cows (Supplementary Fig. 1). Next, PCR gel electrophoresis was conducted on experimental samples derived from experimental calves. The integrity of microbial DNA was tested using gel electrophoresis with a 1% (w/v) agarose gel. The purity and quantity of microbial DNA was evaluated using a spectrophotometer, Eppendorf BioPhotometer D30 (Eppendorf AG, Hamburg, Germany). The 260/280 ratio of 1.8–2.0 confirmed the purity of DNA. Tris acetate EDTA buffer (0.5X) was used for the preparation of the agarose buffer. PCR was performed in 15 μl aliquots consisting of 7.5 μl of PCR Master Mix (Takara Bio Inc., Japan), 2.0 μl of each primer (10 pmol/μl, forward and reverse, Table 4), 6.0 µl of nuclease-free water, and 1.0 µl of DNA template (75 ng/µl). PCR was carried out using the PCR system (Master Cycler, Eppendorf, Germany) under the following conditions (37 cycles): initial denaturation at 94 °C for 120 s, final denaturation at 94 °C for 25 s, annealing at 60 °C for 20 s, extension at 72 °C for 20 s, and final extension72°C for 7 min. In addition, a subset of negative samples (without DNA template but with primers) was used in conjunction with the test samples to determine the specificity of qPCR. Then, a limit (i.e., < 40 Ct) was set on the basis of the Ct observed for the negative sample at each run. Positive controls included a DNA template that was synthesized from fresh rumen fluid samples (from cows); these samples were cultivated on anaerobic culture medium and displayed a band using PCR gel electrophoresis.

### Chemical analysis

The ingredients and the chemical compositions of the calf starters are shown in Table 3. Nutrient analysis of the starter feed was carried out according to AOAC (2016)^65^: dry matter (DM, method 925.40), crude protein (CP, method 2001.11), ether extract (EE, method 920.39), ash (method 942.05), starch (method 996.11), neutral detergent fiber (NDF, using 100 μl/heat resistant alpha-amylase sample, sodium sulfite was used during ND extraction to reduce nitrogenous contamination), and acid detergent fiber (ADF) using an Ankom Fiber Analyzer System (Ankom Technology, Macedon, NY)^66^. Non-fiber carbohydrate (NFC) was calculated as the following formula:

$${\text{NFC}} = 100 - \% {\text{neutral}}\;{\text{detergent}}\;{\text{fiber}} - \% \;{\text{crude}}\;{\text{protein}} - \% \;{\text{ether}}\;{\text{extract}} - \% \;{\text{ash}}.$$

For the determination of large-peptide N (LPep N) and small-peptide plus AA N (SPep + AA N), 30 mg of feed samples were weighed into a glass-stoppered flask and mixed with tungstic acid (TA) and trichloroacetic acid (TCA) as described by Licitra et al.^17^. Then, TA-soluble N (TA N) and TCA-soluble N (TCA N) were used to calculate LPep N and SPep + AA N according to the following formula:

$$\begin{gathered} {\text{LPep N }} = \, \left[ {{\text{TCA}}} \right] \, - \, \left[ {{\text{TA}}} \right] \hfill \\ {\text{SPep }} + {\text{ AA N }} = \, \left[ {{\text{TA}}} \right] \, - \, \left[ {\text{ammonia N}} \right]) \hfill \\ \end{gathered}$$

For ammonia extraction, 20 g samples were put into glass-stoppered flasks, mixed with approximately 60 ml of 2 M KCl, stirred for 30 min, and then filtered through Whatman filter paper (no. 54). NH_3_–N was measured by phenol-hypochlorite assay in the extract ether^67^. For the determination of peptides, five separate samples (i.e., experimental starters, SBM and FSBM) were used.

Table 1 shows the protein content, the CNCPS protein fractions and the peptide and AAs profiles of SBM and FSBM (n = 5, five different feed samples). The values of rumen undegradable protein (RUP) and rumen degradable protein (RDP) were calculated using the equation below (NRC, 2001)^68^:

$${\text{RUP}} = {\text{B}}_{{1}} \left[ {{\text{k}}_{{\text{p}}} /\left( {{\text{k}}_{{\text{d}}} {\text{B}}_{{1}} + {\text{k}}_{{\text{p}}} } \right)} \right] + {\text{B}}_{{2}} \left[ {{\text{k}}_{{\text{p}}} /\left( {{\text{k}}_{{\text{d}}} {\text{B}}_{{2}} + {\text{k}}_{{\text{p}}} } \right)} \right] + {\text{B}}_{{3}} \left[ {{\text{k}}_{{\text{p}}} /\left( {{\text{k}}_{{\text{d}}} {\text{B}}_{{3}} + {\text{k}}_{{\text{p}}} } \right)} \right] + {\text{C}}$$

where k_d_ is the digestion rate of fraction B, and k_p_ is the passage rate of undigested feed.

The AAs content of the feed samples was measured using ion-exchange HPLC with post-column ninhydrin derivatization and fluorescence detection. To this aim, SBM and FSBM samples (n = 5, five separate feed samples) were first hydrolyzed with HCl (containing 0.1% phenol) for 24 h at 110 °C. The resulting chromatograms were incorporated using specialized software (LKB, Biochrom 20; AminoAcid Analyzer, Biotronik GmbH, Maintal, Germany). Cysteine and methionine were analyzed as cysteic acid and methionine sulfone, respectively, by oxidation with performic acid for 16 h at 0 °C. Hydrobromic acid was used after oxidative treatment to consume excess/residual peroxide^69^. For the determination of AAs, five separate samples (i.e., SBM and FSBM) were used.

### Statistical analysis

Data from VFAs and qPCR were analyzed as a completely randomized design with 3 treatments and 13 replications using SAS software; one-way ANOVA followed by multiple comparisons tests, the Tukey’s test. The data was presented as Least Squares Means (LSM) ± Standard Error of the Mean (SEM). Differences between treatments were considered to be significant when *P* < 0.05, although differences between *P* > 0.05 and *P* < 0.10 were considered a tendency towards statistical significance. Orthogonal polynomial contrasts were used to determine the linear or quadratic effect of FSBM using SAS software (SAS Institute Inc., Cary, NC).

Using the Anderson–Darling test, it was confirmed that the data were not normally distributed. As a result, the Spearman method was used to evaluate the potential relationships between each pair of variables, i.e., peptides, ruminal concentrations of NH_3_–N, VFAs and relative abundance of bacteria. The hierarchical clustering analysis (HCA) was conducted with the Spearman distance metric and the unweighted pair group method (UPGMA algorithm). Next, we performed the principal component analysis (PCA) to consider the effects of all variables at the same time. Spearman rho correlation, PCA and network analysis were performed using PAST software (accessible at: https://folk.uio.no/ohammer/past)70.

## Supplementary information

Supplementary Infromation.
