# Supplementary material for: Fermented soybean meal affects the ruminal fermentation and the abundance of selected bacterial species in Holstein calves: a multilevel analysis
Source: Sci Rep. 2020 Jul 21;10:12062. doi: 10.1038/s41598-020-68778-6 (PMC7374609; doi:10.1038/s41598-020-68778-6)
Supplement: Supplementary file 1 — Supplementary Infromation. [file 41598_2020_68778_MOESM1_ESM.pdf]

Fermented soybean meal affects the ruminal fermentation and the abundance of selected bacterial species in Holstein calves: a multilevel analysis

Leila Kaviani Feizi[1], Sabihe Soleymanian Zad[2,3], Seyed Amir Hossein Jalali [3,4], Hassan Rafiee[1], Masoud Boroumand Jazi[5], Khaled Sadeghi[1], Rasoul Kowsar[1]*

[1]Department of Animal Science, College of Agriculture, Isfahan University of Technology, Isfahan 84156-83111, Iran.

[2]Department of Food Science and Technology, College of Agriculture, Isfahan University of Technology, Isfahan 84156-83111, Iran

[3]Research Institute for Biotechnology and Bioengineering, Isfahan University of Technology, 10 Isfahan 84156-83111, Iran

[4]Department of Natural Resources, Isfahan University of Technology, Isfahan 84156 -83111, Iran

[5]Animal Science Research Department, Isfahan Agricultural and Natural Resources Research and Education Center, Esfahan 81846-35745, Iran

*Corresponding author: Rasoul Kowsar, Isfahan University of Technology, E-mail: Rasoul_kowsarzar@yahoo.com

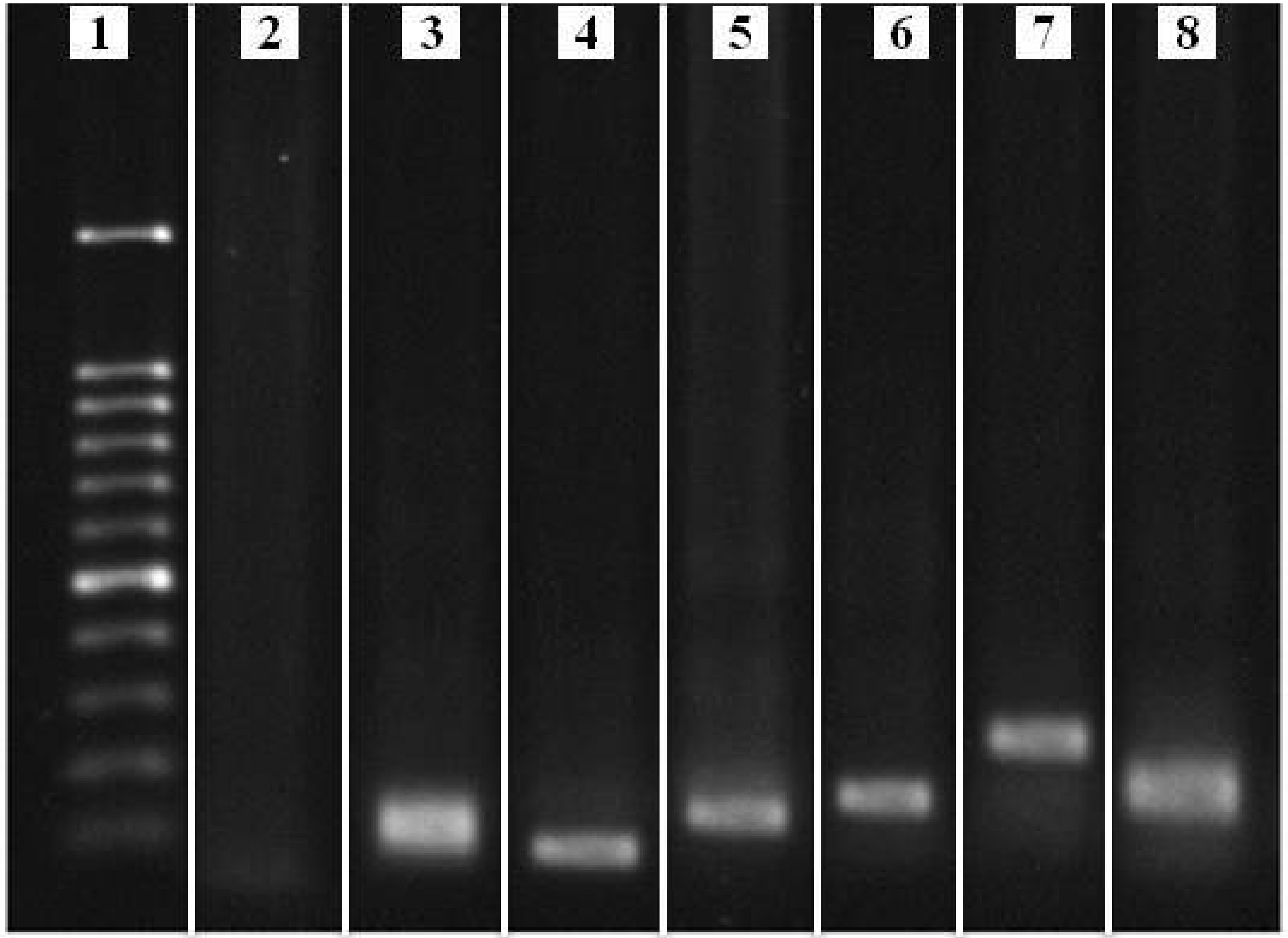

Supplementary Figure 1. Outcomes of conventional PCR.
The gel displays PCR products from cDNA synthesized from:
Lane 1: 100 bp DNA ladder
Lane 2: negative control
Lane 3: *Fibrobacter succinogenes* (121 bp)
Lane 4: *Prevotella ruminicola* (74 bp)
Lane 5: *Ruminococcus flavefaciens* (132 bp)
Lane 6: *Ruminococcus albus* (176 bp)
Lane 7: *Butyrivibrio fibrisolvens* (295 bp)
Lane 8: Total bacteria (192 bp)

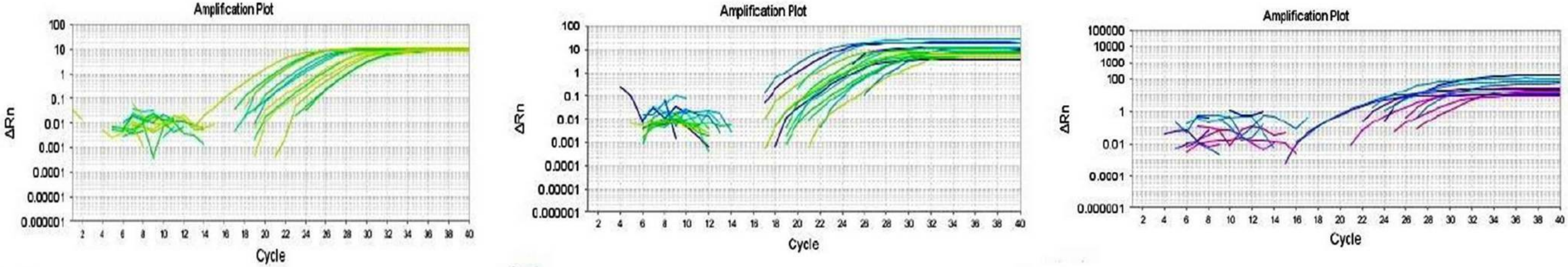

Butyrivibrio fibrisolvens | Prevotella ruminicola | Ruminococcus albus

Supplementary Figure 2. Dissociation curves demonstrate the specificity of the real-time qRT-PCR in the quantification of ruminal bacteria in the calves.

This Figure indicate the dissociation curves for different specific primers for the amplification of rumen bacteria.

PC	Eigenvalue	% variance
1	3.20403	24.646
2	2.42754	18.673
3	2.00189	15.399
4	1.39741	10.749
5	1.10614	8.5088
6	0.909019	6.9925
7	0.741947	5.7073
8	0.430771	3.3136
9	0.290589	2.2353
10	0.254428	1.9571
11	0.172914	1.3301
12	0.038021	0.29247
13	0.025302	0.19463

Supplementary Table 1. The scree plot of the PCA analysis shows that the first four principle axis factors accounted for a sufficient amount of total variance (69.5 %).
